# Supplementary material for: Investigating Polymorphisms and Expression Profile of Immune, Antioxidant, and Erythritol-Related Genes for Limiting Postparturient Endometritis in Holstein Cattle
Source: Vet Sci. 2023 May 23;10(6):370. doi: 10.3390/vetsci10060370 (PMC10305243; doi:10.3390/vetsci10060370)
Supplement: Supplementary file 1 [file vetsci-10-00370-s001.zip › vetsci-2376489-supplementary.pdf]

Supplementary

```
KX138607.1      AGAGACGACACTACAGTGCCTCGGAGGCTGCCCTGGCCGGCACAGACAGAGGGTTATGCT 60
H               AGAGACGACACTACAGTGCCTCGGAGGCTGCCCTGGCCGGCACAGACAGAGGGTCATGCT 60
E               AGAGACGACACTACAGTGCCTCGGAGGCTGCCCTGGCCGGCACAGACAGAGGGTTATGCT 60
                ****************************************************** *****
KX138607.1      TTCACAGAGCCACTTCTGGTCACAGAAAATGCCAGGATGATGGCGCGTGCCCGCCTGGCT 120
H               TTCACAGAGCCACTTCTGGTCACAGAAAATGCCAGGATGATGGCGCGTGCCCGCCTGGCT 120
E               TTCACAGAGCCACTTCTGGTCACAGAAAATGCCAGGATGATGGCGCGTGCCCGCCTGGCT 120
                ************************************************************
KX138607.1      GCGGCTCTGATCCCAGCCACGGCCATCCTCTCCTGCCTGAGAACCGAGAGTTGGGACCCT 180
H               GCGGCTCTGATCCCAGCCACGGCCATCCTCTCCTGCCTGAGAACCGAGAGCTGGGACCCT 180
E               GCGGCTCTGATCCCAGCCACGGCCATCCTCTCCTGCCTGAGAACCGAGAGTTGGGACCCT 180
                *************************************************** ********
KX138607.1      TGCGTACAGGTTGTTCCTAACATTAGTTACCAGTGCATGGAGCTGAATCTCTACAAAATC 240
H               TGCGTACAGGTTGTTCCTAACATTAGTTACCAGTGCATGGAGCTGAATCTCTACAAAATC 240
E               TGCGTACAGGTTGTTCCTAACATTAGTTACCAATGCATGGAGCTGAATCTCTACAAAATC 240
                ******************************** ***************************
KX138607.1      CCCGACAACATCCCCATATCAACCAAGATGCTGGACCTGAGCTTGAACTACCTGAGACAT 300
H               CCCGACAACATCCCCATATCAACCAAGATGCTGGACCTGAGCTTTAACTACCTGAGACAT 300
E               CCCGACAACATCCCCATATCAACCAAGATGCTGGACCTGAGCTTGAACTACCTGAGACAT 300
                ******************************************** ***************
KX138607.1      TTAGGCAGCCATAACTTCTCCAGCTTCCCAGAACTGCAAGTGCTGGATTTATCCAGATGT 360
H               TTAGGCAGCCATAACTTCTCCAGCTTCCCAGAACTGCAAGTGCTGGATTTATCCAGATGT 360
E               TTAGGCAGCCATAACTTCTCCAGCTTCCCAGAACTGCAAGTGCTGGATTTATCCAGATGT 360
                ************************************************************
KX138607.1      GAAATTAAGATTATTGAAGACGACACATTTCAGGGCCTAGACCACCTCTCCACCTTGATA 420
H               GAAATTAAGATTATTGAAGATGACACATTTCAGGGCCTAAACCACCTCTCCACCTTGATA 420
E               GAAATTAAGATTATTGAAGAEGACACATTTCAGGGCCTAGACCACCTCTCCACCTTGATA 420
                ******************** ****************** ********************
KX138607.1      CTGACGGGAAACCCTATCCAGAGCTTAGCCTGGGGAGCCTTTTCTGGGCTATCAAGTTTA 480
H               CTGACGGGAAACCCTATCCAGAGCTTAGCCTGGGGAGCCTTTTCTGGGCTATCAAGTTTA 480
E               CTGACGGGAAACCCTATCCAGAGCTTAGCCTGGGGAGCCTTTTCTGGGCTATCAAGTTTA 480
                ************************************************************
KX138607.1      CAGAAGCTGGCGGCCGTGGAGACAAACCTAGTCTCTCTAAATGACTTC   528
H               CAGAAGCTGGCGGCCGTGGAGACAAACCTAGTCTCTCTAAATGACTTC   528
E               CAGAAGCTGGTGGCCGTGGAGACAAACCTAGTCTCTCTAAATGACTTC   528
                ********** *************************************
```

Figure S1. Assessment of healthy and endometritis dairy cows with sequences derived from GenBank gb|KX138607.1| and *TLR4* marker (528-bp), indicating nitrogenous bases matching for DNA.

```
EF583900.1      TTTTCCACAGCTCATCTCTTCACCTCAGAAGACTCCAGACCTGGACTCACTCCATGCAGC 60
H               TTTTCCACAGCTCATCTCTTCACCTCAGAAGACTCCAGACCTGGACTCACTCCATACAGC 60
E               TTTTCCACAGCTCATCTCTTCACCTCAGAAGACTCCAGACCTGGACTCACTCCATGCAGC 60
                ******************************************************* ****
EF583900.1      AAAGAAAGTTGATGCTACTGGGTCCATCTCAGGCTGATCTTGACACCTCTCATGTTCTGC 120
H               AAAGAAAGTTGATGCTACTGGGTCCATCTCAGGCTGATCTTGACACCTCTCATGTTCTGC 120
E               AAAGAAAGTTGATGCTACTGGGTCCATCTCAGGCTGATCTTGACACCTCTCATGTTCTGC 120
                ************************************************************
EF583900.1      TCTCTTCAACCAGACCTCTCTGTTTCATTTTGGAAGAAGACTGAAAATGGTGTTTCCAAT 180
H               TCTCTTCAACCAGACCTCTCTGTTTCATTTTGGAAGAAGACTGAAAATGGTGTTTCCAAT 180
E               TCTCTTCAACCAGACCTCTCTGTTTCATTTTGGAAGAAGACTGAAAATGGTGTTTCCAAT 180
                ************************************************************
EF583900.1      GTGGACATTGAAGAGACAGTTTCCTATCCTTTTTAACATGATCCTAATTTCTGGACTCCT 240
H               GTGGACATTGAAGAGACAGTTTCCTATCCTTTTTAACATGATCCTAATTTCTGGACTCCT 240
E               GTGGACATTGAAGAGACAGTTTCCTATCCTTTTTAACATGATCCTAATTTCTGGACTCCT 240
                ************************************************************
EF583900.1      TGGGGCTAGATGGTTTCCTAAAACTCTGCCCTGTGATGTCACTCTGGATGCCCCAAATAC 300
H               TGGGGCTAGATGGTTTCCTAAAACTCTGCCCTGTGATGTCACTCTGGATGCCCCAAATAC 300
E               TGGGGCTAGATGGTTTCCTAAAACTCTGCCCTGTGATGTCACTCTGGATGCCCCAAATAC 300
                ************************************************************
EF583900.1      CCATGTGATTGTGGACTGCACAGACAAGCATTTGACAGAAATTCCTGGAGGTATTCCTGC 360
H               CCATGTGATTGTGGACTGCACAGACAAGCATTTGACAGAAATTCCTGGAGGTATTCCTGC 360
E               CCATGTGATTGTGGACTGCACAGACAAGCATTTGACAGAAATTCCTGGAGGTATTCCTGC 360
                ************************************************************
EF583900.1      CAATGCCACCAACCTTACCCTCACCATTAACCACATAGCAGGCATCTCTCCAGCCTCCTT 420
H               CAATGCCACCAACCTTACCCTCACCATTAACCACATAGCAGGCATCTCTCCAGCCTCCTT 420
E               CAATGCCACCAACCTTACCCTCACCATTAACCACATAGCAGGCATCTCTCCAGCCTCCTT 420
                ************************************************************
```

Figure S2. Assessment of healthy and endometritis dairy cows with sequences derived from GenBank gb|EF583900.1| and *TLR7* marker (420-bp), indicating nitrogenous bases matching for DNA.

```
NM_173966.3     ACCAGCCAGGAGAGAGACAAGCAGCTGCAGAACCCCCTGGAGATAACCTCCCAGACAACA     60
H               ACCAGCCAGGAGAGAGACAAGCAGCTGCAGAACCCCCTGGAGATAACCTCCCAGACAACA     60
E               ACCAGCCAGGAGAGAGACAAGCAGCTGCAGAACCCCCTGGAGATAACCTCCCAGACAACA     60
                ************************************************************
NM_173966.3     CACCCCCGAGAGACAGCCAGGCAACTTGCTCTCTCTCACATACCCTGCCACAAGGCTCTC     120
H               CACCCCCGAGAGACAGCCAGGCAACTCGCTCTCTCTCACATACCCTGCCACAAGGCTCTC     120
E               CACCCCCGAGAGACAGCCAGGCAACTTGCTCTCTCTCACATACCCTGCCACAAGGCTCTC     120
                ************************** *********************************
NM_173966.3     CTGTCTCCCGTCTGGACTTGAACCCTTCTGAAAAAGACACCATGAGCACCAAAAGCATGA     180
H               CTGTCTCCCGTCTGGACTTGAACCCTTCTGAAAAAGACACCATGAGCACCAAAAGCATGA     180
E               CTGTCTCCCGTCTGGACTTGAACCCTTCTGAAAAAGACACCATGAGCACCAAAAGCATGA     180
                ************************************************************
NM_173966.3     TCCGGGATGTGGAGCTGGCGGAGGAGGTGCTCTCCGAGAAAGCAGGGGGCCCCCAGGGCT     240
H               TCCGGGATGTGGAGCTGGCGGAGGAGGTGCTCTCCGAGAAAGCAGGGGGCCCCCAGGGCT     240
E               TCCGGGATGTGGAGCTGGCGGAGGAGGCGCTCTCCGAGAAAGCAGGGGGCCCCCAGGGCT     240
                *************************** ********************************
NM_173966.3     CCAGAAGTTGCTTGTGCCTCAGCCTCTTCTCCTTCCTCCTGGTTGCAGGAGCCACCACGC     300
H               CCAGAAGTTGCTTGTGCCTCAGCCTCTTCTCCTTCCTCCTGGTTGCAGGAGCCACCACGC     300
E               CCAGAAGTTGCTTGTGCCTCAGCCTCTTCTCCTTCCTCCTGGTTGCAGGAGCCACCACGC     300
                ************************************************************
NM_173966.3     TCTTCTGCCTGCTGCACTTCGGGGTAATCGGCCCCCAGAGGGAAGAGCAGTCCCCAGGTG     360
H               TCTTCTGCCTGCTGCACTTCGGGGTAATCGGCCCCCAGAGGGAAGAGCAGTCCCCAGGTG     360
E               TCTTCTGCCTGCTGCACTTCGGGGTAATCGGCCCCCAGAGGGAAGAGCAGTCCCCAGGTG     360
                ************************************************************
NM_173966.3     GCCCCTCCATCAACAGCCCTCTGGTTCAAACACTCAGGTCCTCTTCTCAAGCCTCAAGTA     420
H               GCCCCTCCATCAACAGCCCTCTGGTTCAAACACTCAGGTCCTCTTCTCAAGCCTCAAGTA     420
E               GCCCCTCCATCAACAGCCCTCTGGTTCAGACACTCAGGTCCTCTTCTCAAGCCTCAAGTA     420
                **************************** *******************************
NM_173966.3     ACAAGCCGGTAGCCCACGTTGTAGCCGACATCAACTCTCCGGGGCAGCTCCGGTGGTGGG     480
H               ACAAGCCGGTAGCCCACGTTGTAGCCGACATCAACTCTCCGGGGCAGCTCCGGTGGTGGG     480
E               ACAAGCCGGTAGCCCACGTTGTAGCCGACATCAACTCTCCGGGGCAGCTCCGGTGGTGGG     480
                ************************************************************
NM_173966.3     ACTCGTATGCCAATGCCCTCATGGCCAACGGTGTGAAGCTGGAAGACAACCAGCTGGTGG     540
H               ACTCGTATGCCAATGCCCTCATGGCCAACGGTGTGAAGCTGGAAGACAACCAGCTGGTGG     540
E               ACTCGTATGCCAATGCCCTCATGGCCAACGGTGTGAAGCTGGAAGACAACCAGCTGGTGG     540
                ************************************************************

NM_173966.3     TGCCTGCTGAC 551
H               TGCCTGCTGAC 551
E               TGCCTGCTGAC 551
                ***********
```

Figure S3. Assessment of healthy and endometritis dairy cows with sequences derived from GenBank gb|NM_173966.1| and *TNF-α* marker (551-bp), indicating nitrogenous bases matching for DNA.

```
NM_174088.1      ATGCATAGCTCAGCACTACTCTGTTGCCTGGTCTTCCTGGCTGGGGTGGCAGCCAGCCGA
H                ATGCATAGCTCAGCACTACTCTGTTGCCTGGTCTTCCTGGCTGGGGTGGCAGCCAGCCGA
E                ATGCATAGCTCAGCACTACTCTGTTGCCTGGTCTTCCTGGCTGGGGTGGCAGCCAGCCGA
                 ************************************************************
NM_174088.1      GATGCGAGCACCCTGTCTGACAGCAGCTGTATCCACTTGCCAACCAGCCTGCCCCACATG
H                GATGCGAGCACCCTGTCTGACAGCAGCTGTATCCACTTGCCAACCAGCCTGCCCCACATG
E                GATGCGAGCACCCTGTCTGACAGCAGCTGTATCCACTTGCCAACCAGCCTGCCCCACATG
                 ************************************************************
NM_174088.1      CTGCGGGAGCTCCGAGCTGCCTTCGGCGAGGCGAAGACTTTCTTTCAAATGAAGGACCAA
H                CTGCGGGAGCTCCGAGCTGCCTTCGGCGAGGCGAAGACTTTCTTTCAAATGAAGGACCAA
E                CTGCGGGAGCTCCGAGCTGCCTTCGGCAAGGTGAAGACTTTCTTTCAAATGAAGGACCAA
                 *************************** *** ****************************
NM_174088.1      CTGCACAGCTTACTGTTGACCCAGTCTCTGCTGGATGACTTTAAGGGTTACCTGGGTTGC
H                CTGCACAGCTTACTGTTGACCCAGTCTCTGCTGGATGACTTTAAAGGTTACCTGGGTTGC
E                CTGCACAGCTTACTGTTGACCCAGTCTCTGCTGGATGACTTTAAGGGTTACCTGGGTTGC
                 ******************************************** ***************
NM_174088.1      CAAGCCTTGTCGGAAATGATCCAGTTTTACCTGGAAGAGGTGATGCCACAGGCTGAGAAC
H                CAAGCCTTGTCGGAAATGATCCAGTTTTACCTGGAAGAGGTGATGCCACAGGCTGAGAAC
E                CAAGCCTTGTCGGAAATGATCCAGTTTTACCTGGAAGAGGTGATGCCACAGGCTGAGAAC
                 ************************************************************
NM_174088.1      CACGGGCCTGACATCAAGGAGCACGTGAACTCACTGGGGGAGAAGCTGAAGACCCTGCGG
H                CACGGGCCTGACATCAAGGAACACGTGAACTCACTGGGGGAGAAGCTGAAGACCCTGCGG
E                CACGGGCCTGACATCAAGGAGCACGTGAACTCACTGGGGGAGAAGCTGAAGACCCTCCGG
                 ******************** *********************************** ***
NM_174088.1      CTGCGGCTGCGGCGCTGTCATCGCTTTCTGCCCTGCGAAAACAAGAGCAAGGCGGTGGAG
H                CTGCGGCTGCGGCGCTGTCATCGCTTTCTGCCCTGCGAAAACAAGAGCAAGGCGGTGGAG
E                CTGCGGCTGCGGCGCTGTCATCGCTTTCTGCCCTGCGAAAACAAGAGCAAGGCGGTGGAG
                 ************************************************************
NM_174088.1      AAGGTGAAGAGAGTCTTCAGTGAGCTCCAAGAGAGGGGTGTCTACAAAGCCATGAGTGAG
H                AAGGTGAAGAGAGTCTTCAGTGAGCTCCAAGAGAGGGGTGTCTACAAAGCCATGAGTGAG
E                AAGGTGAAGAGAGTCTTCAGTGAGCTCCAAGAGAGGGGTGTCTACAAAGCCATGAGTGAG
                 ************************************************************
NM_174088.1      TTTGACATCTTCATCAACTACATAGAAACCTACATGACAACGAAGATGCAAAAGTGAAGC
H                TTTGACATCTTCATCAACTACATAGAAACCTACATGACAACGAAGATGCAAAAGTGAAGC
E                TTTGACATCTTCATCAACTACATAGAAACCTACATGACAACGAAGATGCAAAAGTGAAGC
                 ************************************************************
NM_174088.1      ATTCTAGGGAAGAAGACCTCCAGGATGGTGA                 571
H                ATTCTAGGGAAGAAGACCTCCAGGATGGTGA                 571
E                ATTCTAGGGAAGAAGACCTCCAGGATGGTGA                 571
                 *******************************
```

Figure S4. Assessment of healthy and endometritis dairy cows with sequences derived from GenBank gb|NM_174088.1| and *IL10* marker (571-bp), indicating nitrogenous bases matching for DNA.

```
NM_001045983.1      CCTGGGACCACAGCCTAAACGAGGCTAGCTGGAGGGAAGTGAGAGGTGACCTTGGTCTGG      60
H                   CCTGGGACCACAGCCTAAACGAGGCTAGCTGGAGGGAAGTGAGAGGTGACCTTGGTCTGG      60
E                   CCTGGGACCACAGCCTAAACGAGGCTAGCTGGAGGGAAGTGAGAGGTGACCTTGGTCTGG      60
                    ************************************************************

NM_001045983.1      GCCCAGTCGTGAGAACCTCCGCCACCCCTGCCCTGGACTGACAGCCATGGCCAAGGCACA      120
H                   GCCCAGTCGTGAGAACCTCCGCCACCCCTGCCCTGGACTGACAGCCATGGCCAAGGCACA      120
E                   GCCCAGTCGTGAGAACCTCCGCCACCCCTGCCCTGGACTGACAGCCATGGCCAAGGCACA      120
                    ************************************************************

NM_001045983.1      GCAACTTCGGGCTGAGAGCGACTTCGACCAGCTTCCTGACGACATTGCCATCTCAGCCAA      180
H                   GCAACTTCGGGCTGAGAGCGACTTCGACCAGCTTCCTGACGACATTGCCATCTCAGCCAA      180
E                   GCAACTTCGGGCTGAGAGCGACTTCGACCAGCTTCCTGACGACATTGCCATCTCAGCCAA      180
                    ************************************************************

NM_001045983.1      CATCGCTGACATTGAGGAGAAAAGAGGCTTCACCAGCCACTTTGTTTTTGTCATCGAGGT      240
H                   CATCGCTGACATTGAGGAGAAAAGAGGCTTCACCAGCCACTTTGTTTTTGTCATCGAGGT      240
E                   CATCGCTGACATTGAGGAGAAAAGAGGCTTCACCAGCCACTTTGTTTTTGTCATCGAGGT      240
                    ************************************************************

NM_001045983.1      AAAGACGAAAGGGGGGTCCAAGTACCTCATCTACCGCCGCTACCGCCAGTTCTACGCCTT      300
H                   AAAGACGAAAGGGGGGTCCAAGTACCTCATCTACCGCCGCTACCGCCAGTTCTACGCCTT      300
E                   AAAGACGAAAGGGGGGTCCAAGTACCTCATCTACCGCCGCTACCGCCAGTTCTACGCCTT      300
                    ************************************************************

NM_001045983.1      ACAGAGCAAGCTGGAGGAGCGCTTCGGTCAGGAGAGCAAGACCAGCCCCTTAACCTGTAT      360
H                   ACAGAGCAAGCTGGAGGAGCGCTTCGGTCAGGAGAGCAAGACCAGCCCCTTAACCTGTAT      360
E                   ACAGAGCAAGCTGGAGGAGCGCTTCGGTCAGGAGAGCAAGACCAGCCCCTTAACCTGTAT      360
                    ************************************************************

NM_001045983.1      CCTCCCCACGCTCCCAGCCAAAGTCTACGTGGGTGTGAAACAGGAGATTGCCGAGATGCG      420
H                   CCTCCCCACGCTCCCAGCCAAAGTCTACGTGGGTGTGAAACAGGAGATTGCCGAGATGCG      420
E                   CCTCCCCACGCTCCCAGCCAAAGTCTACGTGGGTGTGAAACAGGAGATTGCCGAGATGCG      420
                    ************************************************************

NM_001045983.1      AATACCTGCCCTCAACGCCTACATGAAGCACCTCCTCAGCCTGCCCATCTGGGTGCTGAT      480
H                   AATACCTGCCCTCAACGCCTACATGAAGCACCTCCTCAGCCTGCCCATCTGGGTGCTGAT      480
E                   AATACCTGCCCTCAACGCCTACATGAAGCACCTCCTCAGCCTGCCCATCTGGGTGCTGAT      480
                    ************************************************************

NM_001045983.1      GGACGAGGACGTTCGCATCTTCTTCTACCAGTCGTCCTACGACGCCGAGCAGGTGCCTCA      540
H                   GGACGAGGACGTTCGCATCTTCTTCTACCAGTCGTCCTACGACGCCGAGCAGGTGCCTCA      540
E                   GGACGAGGACGTTCGCATCTTCTTCTACCAGTCGTCCTACGACGCCGAGCAGGTGCCTCA      540
                    ************************************************************

NM_001045983.1      AGCGCTCCGGCGGCTCCGCCCGCGCACCCGGCGAGTAAAAAGCGAGTCCCCACAAGCTGC      600
H                   AGCGCTCCGGCGGCTCCGCCCGCGCACCCGGCGAGTAAAAAGCGAGTCCCCACAAGCTGC      600
E                   AGCGCTCCGGCGGCTCCGCCCGCGCACCCGGCGAGTAAAAAGCGAGTCCCCACAAGCTGC      600
                    ************************************************************

NM_001045983.1      TGGCATTGACCGCATGGCAGCTCCACGAGCAGAGGCCCTGTTTGATTTCACTGGGAACAG      660
H                   TGGCATTGACCGCATGGCAGCTCCACGAGCAGAGGCCCTGTTTGATTTCACTGGGAACAG      660
E                   TGGCATTGACCGCATGGCAGCTCCACGAGCAGAGGCCCTGTTTGATTTCACTGGGAACAG      660
                    ************************************************************

NM_001045983.1      CAAACATGAGCTGAATTTCAAAGTTGGAGATGTGATCTTCCTTCTCAGTCGGATCAATAA      720
H                   CAAACATGAGCTGAATTTCAAAGTTGGAGATGTGATCTTCCTTCTCAGTCGGATCAATAA      720
E                   CAAACATGAGCTGAATTTCAAAGTTGGAGATGTGATCTTCCTTCTCAGTCGGATCAATAA      720
                    ************************************************************

NM_001045983.1      AGACTGGCTGGAGGGCACTGTCCAGGGAACCACAGGCATCTTCCCAGTGTCCTTTGTGAA      780
H                   AGACTGGCTGGAGGGCACTGTCCAGGGAACCACAGGCATCTTCCCAGTGTCCTTTGTGAA      780
E                   AGACTGGCTGGAGGGCACTGTCCGGGGAACCACAGGCATCTTCCCAGTGTCCTTTGTGAA      780
                    *********************** ************************************

NM_001045983.1      GATCCTCAAGGACTTCCCAGAGGAGGAAGACCCCACCAACTGGCTACGCTGCTATTACTA      840
H                   GATCCTCAAGGACTTCCCAGAGGAGGAAGACCCCACCAACTGGCTACGCTGCTATTACTA      840
E                   GATCCTCAAGGACTTCCCAGAGGAGGAAGACCCCACCAACTGGCTACGCTGCTATTACTA      840
                    ************************************************************

NM_001045983.1      TGAGGACACCATCAGCACCATCAAG   865
H                   TGAGGACACCATCAGCACCATCAAG   865
E                   TGAGGACACCATCAGCACCATCAAG   865
                    *************************
```

Figure S5. Assessment of healthy and endometritis dairy cows with sequences derived from GenBank gb|NM_00145983.1| and *NCF4* marker (865-bp), indicating nitrogenous bases matching for DNA.

```
NM_001046252.2      CTTTCTATGAAGGGCTTTTTTCTGGTAGAGTGATTGTTTTTTATATTAACACTTTTTGTG      60
H                   CTTTCTATGAAGGGCTTTTTTCTGGTAGAGTGATTGTTTTTTATATTAACACTTTTTGTG      60
E                   CTTTCTATGAAGGGCTTTTTTCTGGTAGAGTGATTGTTTTTTATATTAACACTTTTTGTG      60
                    ************************************************************

NM_001046252.2      ACCTTCTTTTGGTCTTAAACACCTTTGAGCATAATTTTGAAAGAAGGGACTTACAGAACC      120
H                   ACCTTCTTTTGGTCTTAAACACCTTTGAGCATAATTTTGAAAGAAGGGACTTACAGAACC      120
E                   ACCTTCTTTTGGTCTTAAACACCTTTGAGCATAATTTTGAAAGAAGGGACTTACAGAACC      120
                    ************************************************************

NM_001046252.2      ATTATCCATAACCATATGACTGGTATCATCTTCCTATTTGCCAGTGCACACTCACTTATT      180
H                   ATTATCCATAACCATATGACTGGTATCATCTTCCTATTTGCCAGTGCACACTCACTTATT      180
E                   ATTATCCATAACCATATGACTGGTATCATCTTCCTATTTGCCAGTGCACACTCACTTATT      180
                    ************************************************************

NM_001046252.2      TTTTTTTTTTTGCCTTAACAGGAAATGTAATGGATAATTTCATCCACTGCCTTTTCTTGG      240
H                   TTTTTTTTTTTGCCTTAACAGGAAATGTAATGGATAATTTCATCCACTGCCTTTTCTTGG      240
E                   TTTTTTTTTTTGCCTTAACAGGAAATGTAATGGATAATTTCATCCACTGCCTTTTCTTGG      240
                    ************************************************************

NM_001046252.2      GTTTAGTGTGTTTCCTAGAAACCTCTATAATGGAACTCATTAAAGTTGGGCCCTTTTGGG      300
H                   GTTTAGTGTGTTTCCTAGAAACCTCTATAATGGAACTCATTAAAGTTGGGCCCTTTTGGG      300
E                   GTTTAGTGTGTTTCCTAGAAACCTCTATAATGGAACTCATTAAAGTTGGGCCCTTTTGGG      300
                    ************************************************************

NM_001046252.2      GTTCCTAATGGATGTTTGGAAAGACAAAGCACGTTTGAGCACGTCTGGGTACCATGGTGC      360
H                   GTTCCTAATGGATGTTTGGAAAGACAAAGCACGTTTGAGCACGTCTGGGTACCATGGTGC      360
E                   GTTCCTAATGGATGTTTGGAAAGACAAAGCACGTTTGAGCACGTCTGGGTACCATGGTGC      360
                    ************************************************************

NM_001046252.2      TGAAAAGCGTGGGGACCCGACTGTTTCAAAGACCTCTGAAGTCTGGATTTTATTTTTCAG      420
H                   TGAAAAGCGTGGGGACCCGACTGTTTCAAAGGCCTCTGAAGTCTGGATTTTATTTTTCAG      420
E                   TGAAAAGCGTGGGGACCCGACTGTTTCAAAGACCTCTGAAGTCTGGATTTTATTTTTCAG      420
                    ******************************* ****************************

NM_001046252.2      AAATACAGTTTGTTTGTATAACATCGGAAGACATGATTCCTAGGACTGAAGCAGCAAGCT      480
H                   AAATACAGTTTGTTTGTATAACATCGGAAGACATGATTCCTAGGACTGAAGCAGCAAGCT      480
E                   AAATACAGTTTGTTTGTATAACATCGGAAGACATGATTCCTAGGACTGAAGCAGCAAGCT      480
                    ************************************************************

NM_001046252.2      AGAGTTCTCAGTTCGCTGCTGTGTCATCTTTGAAATCAAGACAAAGCCGGGCTGGACTTT      540
H                   AGAGTTCTCAGTTCGCTGCTGTGTCATCTTTGAAATCAAGACAAAGCCGGGCTGGACTTT      540
E                   AGAGTTCTCAGTTCGCTGCTGTGTCATCTTTGAAATCAAGACAAAGCCGGGCTGGACTTT      540
                    ************************************************************

NM_001046252.2      CAAGAGTCCCTGTTTTGATAATGAATAGGCACAAGGAATGCATGTGAATAATACTGTGTA      600
H                   CAAGAGTCCCTGTTTTGATAATGAATAGGCACAAGGAATGCATGTGAATAATACTGTGTA      600
E                   CAAGAGTCCCTGTTTTGATAATGAATAGGCACAAGGAATGCATGTGAATAATACTGTGTA      600
                    ************************************************************

NM_001046252.2      GAAATCAAAGCCTAGACATTGATCAAAAATCTGTATATTGGGTG   644
H                   GAAATCAAAGCCTAGACATTGATCAAAAATCTGTATATTGGGTG   644
E                   GAAATCAAAGCCTAGACATTGATCAAAAATCTGTATATTGGGTG   644
                    ********************************************
```

Figure S6. Assessment of healthy and endometritis dairy cows with sequences derived from GenBank gb|NM_001046252.2| and *LITAF* marker (644-bp), indicating nitrogenous bases matching for DNA.

```
XM_005209648.4      GCTCCTGTGGCGTGCACACCCGGAAGCGTGGCCGCGTCAGCGCTCAGAGCCGGTGGAGGC      60
H                   GCTCCTGTGGCGTGCACACCCGGAAGCGTGGCCGCGTCAGCGCTCAGAGCCGGTGGAGGC      60
E                   GCTCCTGTGGCGTGCACACCCGGAAGCGTGGCCGCGTCAGCGCTCAGAGCCGGTGGAGGC      60
                    ************************************************************

XM_005209648.4      GTAGTCACCGCCGCAGCCACCACCTCCTCCTCAATCATGCCGAAGCACGAGTTCTCCGTG      120
H                   GTAGTCACCGCCGCAGCCACCACCTCCTCCTCAATCATGCCGAAGCACGAGTTCTCCGTG      120
E                   GTAGTCACCGCCGCGGCCACCACCTCCTCCTCAATCATGCCGAAGCACGAGTTCTCCGTG      120
                    ************** *********************************************

XM_005209648.4      GATATGACCTGTGAAGGCTGCTCTAACGCAGTCACTCGAGTCCTCAACAAGCTAGGAGGA      180
H                   GATATGACCTGTGAAGGCTGCTCTAACGCAGTCACTCGAGTCCTCAACAAGCTAGGAGGA      180
E                   GATATGACCTGTGAAGGCTGTTCTAACGCAGTCACTCGAGTCCTCAACAAGCTAGGAGGA      180
                    ******************** ***************************************

XM_005209648.4      GTTCAATTTGACATTGACCTGCCCAACAAAAAGGTCTGTATCAACTCTGAGCACAGCGTG      240
H                   GTTCAATTTGACATTGACCTGCCCAACAAAAAGGTCTGTATCAACTCTGAGCACAGCGTG      240
E                   GTTCAATTTGACATTGACCTGCCCAACAAAAAGGTCTGTATCAACTCTGAGCACAGCGTG      240
                    ************************************************************

XM_005209648.4      GACACTTTGCTGGAGACCCTGGGGAAAACAGGAAAGGCTGTCTCCTACCTTGGCCCCAAA      300
H                   GACACTTTGCTGGAGACCCTGGGGAAAACAGGAAAGGCTGTCTCCTACCTTGGCCCCAAA      300
E                   GACACTTTGCTGGAGACCCTGGGGAAAACAGGAAAGGCTGTCTCCTACCTTGGCCCCAAA      300
                    ************************************************************

XM_005209648.4      TAGAGAGGCCCCGTCCAGCAGCCTGTAGGATGGACCAGCATGGGCAGGGCTTTGGGGAAG      360
H                   TAGAGAGGCCCCGTCCAGCAGCCTGTAGGATGGACCAGCATGGGCAGGGCTTTGGGGAAG      360
E                   TAGAGAGGCCCCGTCCAGCAGCCTGTAGGATGGACCAGCATGGGCAGGGCTTTGGGGAAG      360
                    ************************************************************

XM_005209648.4      AAGACCTGGCTGGTTGTCCAGTCCAATGTCCACACACACCCACCCTGCCTCCCCCCGACA      420
H                   AAGACCTGGCTGGTTGTCCAGTCCAATGTCCACACACACCCACCCTGCCTCCCCCCGACA      420
E                   AAGACCTGGCTGGTTGTCCAGTCCAATGTCCACACACACCCACCCTGCCTCCCCCCGACA      420
                    ************************************************************

XM_005209648.4      GTGGGGCCCCAAGTCTTGGCCTGCACACCA      450
H                   GTGGGGCCCCAAGTCTTGGCCTGCACACCA      450
E                   GTGGGGCCCCAAGTCTTGGCCTGCACACCA      450
                    ******************************
```

Figure S7. Assessment of healthy and endometritis dairy cows with sequences derived from GenBank gb|XM_005209648.4| and *ATOX1* marker (450-bp), indicating nitrogenous bases matching for DNA.

```
X61233.1      CGGCTCAGGCCGCCGCCGAGCGCGCTGGAACTTTGCTGCCGCCGCCACCTTTACCGACTT   60
H             CGGCTCAGGCCGCCGCCGAGCGCGCTGGAGCTTTGCTGCCGCCGCCACCTTTACCGACTT   60
E             CGGCTCAGGCCGCCGCCGAGCGCGCTGGAACTTTGCTGCCGCCGCCACCTTTACCGACTT   60
              ***************************** ******************************

X61233.1      CCCCGACTCCAGGATGCCTCCCTACACCATCGTCTACTTCCCGGTTCAAGGGCGCTGCGA   120
H             CCCCGACTCCAGGATGCCTCCCTACACCATCGTCTACTTCCCGGTTCAAGGGCGCTGCGA   120
E             CCCCGACTCCAGGATGCCTCCCTACACCATCGTCTACTTCCCGGTTCAAGGGCGCTGCGA   120
              ************************************************************

X61233.1      GGCCATGCGCATGCTGCTGGCCGACCAGGGCCAGAGCTGGAAGGAGGAGGTCGTAGCCAT   180
H             GGCCATGCGCATGCTGCTGGCCGACCAGGGCCAGAGCTGGAAGGAGGAGGTCGTAGCCAT   180
E             GGCCATGCGCATGCTGCTGGCCGACCAGGGCCAGAGCTGGAAGGAGGAGGTCGTAGCCAT   180
              ************************************************************

X61233.1      GCAGAGCTGGCTGCAGGGCCCACTCAAGGCCTCCTGCCTGTACGGGCAGCTCCCCAAGTT   240
H             GCAGAGCTGGCTGCAGGGCCCACTCAAGGCCTCCTGCCTGTACGGGCAGCTCCCCAAGTT   240
E             GCAGAGCTGGCTGCAGGGCCCACTCAAGGCCTCCTGCCTGTACGGGCAGCTCCCCAAGTT   240
              ************************************************************

X61233.1      CCAGGACGGAGACCTCACGCTGTACCAGTCCAATGCCATCCTGCGGCACCTGGGCCGCAC   300
H             CCAGGACGGAGACCTCACGCTGTACCAGTCCAATGCCATCCTGCGGCACCTGGGCCGCAC   300
E             CCAGGACGGAGACCTCACGCTGTACCAGTCCAATGCCATCCTGCGGCACCTGGGCCGCAC   300
              ************************************************************

X61233.1      CCTCGGGCTGTATGGGAAGGACCAGCAGGAGGCGGCCCTGGTGGACATGGTGAATGACGG   360
H             CCTCGGGCTGTATGGGAAGGACCAGCAGGAGGCGGCCCTGGTGGACATGGTGAATGACGG   360
E             CCTCGGGCTGTATGGGAAGGACCAGCAGGAGGCGGCCCTGGTGGACATGGTGAATGACGG   360
              ************************************************************

X61233.1      TGTAGAGGACCTTCGCTGCAAATACGTCTCCCTCATTTACACCAACTACGAGGCGGGCAA   420
H             TGTAGAGGACCTTCGCTGCAAATACGTCTCCCTCATTTACACCAACTACGAGGCGGGCAA   420
E             TGTAGAGGACCTTCGCTGCAAATACGTCTCCCTCATTTACACCAACTACGAGGCGGGCAA   420
              ************************************************************

X61233.1      GGAGGACTATGTGAAGGCGCTGCCCCAGCACCTGAAGCCTTTCGAGACCCTGCTGTCCCA   480
H             GGAGGACTATGTGAAGGCGCTGCCCCAGCACCTGAAGCCTTTCGAGACCCTGCTGTCCCA   480
E             GGAGGACTATGTGAAGGCGCTGCCCCAGCACCTGAAGCCTTTCGAGACCCTGCTGTCCCA   480
              ************************************************************
```

Figure S8. Assessment of healthy and endometritis dairy cows with sequences derived from GenBank gb|X61233.1| and *GST* marker (480-bp), indicating nitrogenous bases matching for DNA.

```
NM_001075892.2      AGCGCCAGGCGCCGTCCGACCCGTGGCTGTTCGCGAGGCGGTTGGTGGGGGCGCGGCGGC      60
H                   AGCGCCAGGCGCCGTCCGACCCGTGGCTGTTCGCTAGGCGGTTGGTGGGGGCGCGGCGGC      60
E                   AGCGCCAGGCGCCGTCCGACCCGTGGCTGTTCGCGAGGCGGTTGGTGGGGGCGCGGCGGC      60
                    ********************************** *************************

NM_001075892.2      AGCGGTTGGGGGTGGGGAGAGGTGCGGCCAGGAGAAGAGAGAGGTCAGTGAGTTTGCGGG      120
H                   AGCGGTTGGGGGTGGGGAGAGGTGCGGCCAGGAGAAGAGAGAGGTCAGTGAGTTTGCGGG      120
E                   AGCGGTTGGGGGTGGGGAGAGGTGCGGCCAGGAGAAGAGAGAGGTCAGTGAGTTTGCGGG      120
                    ************************************************************

NM_001075892.2      AAGACCGAGTGTCGTGCCGCCGTCATGACCGAGGACTCCAGTGCCCTGCCCTGGTCCATC      180
H                   AAGACCGAGTGTCGTGCCGCCGTCATGACCGAGGACTCCAGTGCCCTGCCCTGGTCCATC      180
E                   AAGACCGAGTGTCGTGCCGCCGTCATGACCGAGGACTCCAGTGCCCTGCCCTGGTCCATC      180
                    ************************************************************

NM_001075892.2      AACAGGGACGATTACGAGCTGCAGGAGGTGATAGGGAGTGGAGCAACGGCTGTGGTCCAA      240
H                   AACAGGGACGATTATGAGCTGCAGGAGGTGATAGGGAGTGGAGCAACGGCTGTGGTCCAA      240
E                   AACAGGGACGATTACGAGCTGCAGGAGGTGATAGGGAGTGGAGCAACGGCTGTGGTCCAA      240
                    ************** *********************************************

NM_001075892.2      GCAGCGTATTGTGCCCCTAAAAAGGAGAAAGTGGCAATCAAACGGATAAACCTTGAGAAA      300
H                   GCAGCGTATTGTGCCCCTAAAAAGGAGAAGGTGGCAATCAAACGGATAAACCTTGAGAAA      300
E                   GCAGCGTATTGTGCCCCTAAAAAGGAGAAAGTGGCAATCAAACGGATAAACCTTGAGAAA      300
                    ***************************** ******************************

NM_001075892.2      TGTCAGACTAGCATGGATGAACTCCTGAAAGAAATCCAAGCCATGAGTCAGTGCCATCAC      360
H                   TGTCAGACTAGCATGGATGAACTCCTGAAAGAAATCCAAGCCATGAGTCAGTGCCATCAC      360
E                   TGTCAGACTAGCATGGATGAACTCCTGAAAGAAATCCAAGCCATGAGTCAGTGCCATCAC      360
                    ************************************************************

NM_001075892.2      CCTAATATTGTGTCTTACTACACATCTTTTGTGGTAAAAGATGAGCTGTGGCTCGTCATG      420
H                   CCTAATATTGTGTCTTACTACACATCTTTTGTGGTAAAAGATGAGCTGTGGCTCGTCATG      420
E                   CCTAATATTGTGTCTTACTACACATCTTTTGTGGTAAAAGATGAGCTGTGGCTAGTCATG      420
                    ***************************************************** ******

NM_001075892.2      AAGCTGTTAAGTGGAGGTTCTGTTCTAGATATTATTAAGCACATTGTGGCAAAGGGGGAA      480
H                   AAGCTGTTAAGTGGAGGTTCTGTTCTAGATATTATTAAGCACATTGTGGCAAAGGGGGAA      480
E                   AAGCTGTTAAGTGGAGGTTCTGTTCTAGATATTATTAAGCACATTGTGGCAAAGGGGGAA      480
                    ************************************************************

NM_001075892.2      CATAAAAATGGGGTCCTGGATGAAGCTACCATCGCTACAATACTC  525
H                   CATAAAAATGGGGTCCTGGATGAAGCTACCATCGCTACAATACTC  525
E                   CATAAAAATGGGGTCCTGGATGAAGCTACCATCGCTACAATACTC  525
                    *********************************************
```

Figure S9. Assessment of healthy and endometritis dairy cows with sequences derived from GenBank gb|NM_001075892.2| and *OXSR1* marker (525-bp), indicating nitrogenous bases matching for DNA.

```
NM_001003906.1      GCTGCTTGCAGCTCCGCAGCCCTGCGTCTTAGCTTTCCAGTCTGCGTCCTCTGCCTCGCC      60
H                   GCTGCTTGCAGCTCCGCAGCCCTGCGTCTTAGCTTTCCAGTCTGCGTCCTCTGCCTCGCC      60
E                   GCTGCTTGCAGCTCCGCAGCCCTGCGTCTTAGCTTTCCAGTCTGCGTCCTCTGCCTCGCC      60
                    ************************************************************

NM_001003906.1      ACCCGCCCGTTCGCCGGGCTGCGCCATGGAGGCCTACCATAAGCCTGATCAGCAGAAGCT      120
H                   ACCCGCCCGTTCGCCGGGCTGCGCCATGGAGGCCTACCATAAGCCTGATCAGCAGAAGCT      120
E                   ACCCGCCCGTTCGCCTGGCTGCGCCATGGAGGCCTACCATAAGCCTGATCAGCAGAAGCT      120
                    *************** ********************************************

NM_001003906.1      GCAGGCCCTGAAGGACACGGCCAACCGCCTGCGTATCAGCTCCATCCAAGCCACCACGGC      180
H                   GCAGGCCCTGAAGGACACGGCCAACCGCCTGCGTATCAGCTCCATCCAAGCCACCACGGC      180
E                   GCAGGCCCTGAAGGACACGGCCAACCGCCTGCGTATCAGCTCCATCCAAGCCACCACGGC      180
                    ************************************************************

NM_001003906.1      GGCCGGATCGGGCCACCCCACGTCATGCTGCAGCGCTGCTGAGATCATGGCTGTCCTCTT      240
H                   GGCCGGATCGGGCCACCCCACGTCATGCTGCAGCGCTGCTGAGATCATGGCTGTCCTCTT      240
E                   GGCCGGATCGGGCCACCCCACGTCATGCTGCAGCGCTGCTGAGATCATGGCTGTCCTCTT      240
                    ************************************************************

NM_001003906.1      CTTCCACACCATGCGCTACAAGGCCCTGGATCCCCGGAACCCTCACAACGACCGCTTTGT      300
H                   CTTCCACACCATGCGCTACAAGGCCCTGGATCCCCGGAACCCTCACAACGACCGCTTTGT      300
E                   CTTCCACACCATGCGCTACAAGGCCCTGGATCCCCGGAACCCTCACAACGACCGCTTTGT      300
                    ************************************************************

NM_001003906.1      TCTCTCCAAGGGCCATGCGGCACCCATCCTGTATGCCGTCTGGGCTGAAGCTGGCTTCCT      360
H                   TCTCTCCAAGGGCCATGCGGCACCCATCCTGTATGCCGTCTGGGCTGAAGCTGGCTTCCT      360
E                   TCTCTCCAAGGGCCATGCGGCACCCATCCTGTATGCCGTCTGGGCTGAAGCTGGCTTCCT      360
                    ************************************************************

NM_001003906.1      GCCGGAGTCAGAGCTGCTGAATCTGAGGAAGATCAACTCCGACTTGGACGGGCACCCTGT      420
H                   GCCGGAGTCAGAGCTGCTGAATCTGAGGAAGATCAGCTCCGACTTGGACGGGCACCCTGT      420
E                   GCCGGAGTCAGAGCTGCTGAATCTGAGGAAGATCAACTCCGACTTGGACGGGCACCCTGT      420
                    *********************************** ************************

NM_001003906.1      CCCGAAACAAGCCTTCACCGATGTGGCCACTGGCTC  456
H                   CCCGAAACAAGCCTTCACCGATGTGGCCACTGGCTC  456
E                   CCCGAAACAAGCCTTCACCGATGTGGCCACTGGCTC  456
                    ************************************
```

Figure S10. Assessment of healthy and endometritis dairy cows with sequences derived from GenBank gb|NM_001003906.1| and *TKT* marker (456-bp), indicating nitrogenous bases matching for DNA.

```
NM_001035433.2      CCACGTGCAGTTGCCGGGACGTTCGCAGTTCGGCGCTGGCAGCACAAGTACCGGCGTCAG      60
H                   CCACGTGCAGTTGCCGGGACGTTCGCAGTTCGGCGCTGGCAGCACAAGTACCGGCATCAG      60
E                   CCACGTGCAGTTGCCGGGACGTTCGCAGTTCGGCGCTGGCAGCACAAGTACCGGCGTCAG      60
                    ******************************************************* ****

NM_001035433.2      GGAGACTAACATCGTCTGTCCGGGCCCGTCCGCGATGTCAAAGGCCGAGGAGGCCAAGAA      120
H                   GGAGACTAACACCGTCTGTCCGGGCCCGTCCGCGATGTCAAAGGCCGAGGAGGCCAAGAA      120
E                   GGAGACTAACATCGTCTGTCCGGGCCCGTCCGCGATGTCAAAGGCCGAGGAGGCCAAGAA      120
                    *********** ************************************************

NM_001035433.2      GCTGGCTGGCCGCGCGGCCGTGGAGAACCACGTGAGGAATAACCAAGTGCTGGGAATTGG      180
H                   GCTGGCTGGCCGCGCGGCCGTGGAGAACCACGTGAGGAATAACCAAGTGCTGGGAATTGG      180
E                   GCTGGCTGGCCGCGCGGCCGTGGAGAACCACGTGAGGAATAACCAAGTGCTGGGAATTGG      180
                    ************************************************************

NM_001035433.2      GAGTGGTTCTACAATTGTCCACGCTGTGCAGCGAATAGCTGAAAGAGTGGAACAAGAGAA      240
H                   GAGTGGTTCTACAATTGTCCACGCTGTGCAGCGAATAGCTGAAAGAGTGGAACAAGAGAA      240
E                   GAGTGGTTCTACAATTGTCCATGCTGTGCAGCGAATAGCTGAAAGAGTGGAACAAGAGAA      240
                    ********************* **************************************

NM_001035433.2      TCTGAAGCTCGTCTGTATTCCCACCTCCTTCCAGGCCCGTCAACTCATCCTGCAGTATGG      300
H                   TCTGAAGCTCGTCTGTATTCCCACCTCCTTCCAGGCCCGTCAACTCATCCTGCAGTATGG      300
E                   TCTGAAGCTCGTCTGTATTCCCACCTCCTTCCAGGCCCGTCAACTCATCCTGCAGTATGG      300
                    ************************************************************

NM_001035433.2      CTTAACTCTCAGTGACCTGGACCGACACCCAGAGATCGATCTTGCCATCGATGGTGCTGA      360
H                   CTTAACTCTCAGTGACCTGGACCGACACCCAGAGATCGATCTTGCCATCGATGGTGCTGA      360
E                   CTTAACTCTCAGTGACCTGGACCGACACCCAGAGATCGATCTTGCCATCGATGGTGCTGA      360
                    ************************************************************

NM_001035433.2      TGAAGTCGACGCTGACCTCAACCTCATCAA       390
H                   TGAAGTCGACGCTGACCTCAACCTCATCAA       390
E                   TGAAGTCGACGCTGACCTCAACCTCATCAA       390
                    ******************************
```

Figure S11. Assessment of healthy and endometritis dairy cows with sequences derived from GenBank gb|NM_001035433.2| and *RPIA* marker (390-bp), indicating nitrogenous bases matching for DNA.

```
NM_001100349.1      CAGAGAGTGCAGATCACTGGCGATTATGCCTCTGGGGTTACAGTTGAAGATTTTGAAATG      60
H                   CAGAGAGTGCAGATCACTGGCGATTATGCCTCTGGGGTTACAGTTGAAGATTTTGAAATG      60
E                   CAGAGAGTGCAGATCACTGGCGATTATGCCTCTGGGGTTACAGTTGAAGATTTTGAAATG      60
                    ************************************************************

NM_001100349.1      GTTTGCAAAGGTCTTTATCGGGCATTGTGTATACGGGAGAAGTATATGCAGAAGTCATTT      120
H                   GTTTGCAAAGGTCTTTATCGGGCATTGTGTATACGGGAGAAGTATATGCAGAAGTCATTT      120
E                   GTTTGCAAAGGTCTTTATCGGGCATTGTGTATACGGGAGAAGTATATGCAGAAGTCATTT      120
                    ************************************************************

NM_001100349.1      CAGAGGTTTCCAAAAACCCCTTCCAAGTACCTAAGGAACATTGATGGTGAGGCTTGGGTA      180
H                   CAGAGGTTTCCAAAAACCCCTTCCAAGTACCTAAGGAACATTGATGGTGAGGCTTGGGTA      180
E                   CAGAGGTTTCCAAAAACCCCTTCCAAGTACCTAAGGAACATTGATGGTGAGGCTTGGGTA      180
                    ************************************************************

NM_001100349.1      GAAAAAGAGAACTTCTATCCAGTCTTTACCCCTCCTATGAAGAAGGGAGAAGACCCTTTC      240
H                   GAAAAAGAGAACTTCTATCCAGTCTTTACCCCTCCTATGAAGAAGGGAGAAGACCCTTTC      240
E                   GAAAAAGAGAACTTCTATCCAGTCTTTACCCCTCCTATGAAGAAGGGAGAAGACCCTTTC      240
                    ************************************************************

NM_001100349.1      CGAACAGATAACCTCCCAGAAAACCTGGGTTATCAGCTCAAAATGAAGGACGGTGTGGTT      300
H                   CGAACAGATAACCTCCCAGAAAACCTGGGTTATCAGCTCAAAATGAAGGACGGTGTGGTT      300
E                   CGAACAGATAACCTCCCAGAAAACCTGGGTTATCAGCTCAAAATGAAGGACGGTGTGGTT      300
                    ************************************************************

NM_001100349.1      TATGTCTATCCTAATGAAGAAGCAGCCAGCAAAGATGAGCCCAAGCCACTTCCTTACCCA      360
H                   TATGTCTATCCTAACGAAGAAGCAGCCAGCAAAGATGAGCCCAAGCCACTTCCTTACCCA      360
E                   TATGTCTATCCTAATGAAGAAGCAGCCAGCAAAGATGAGCCCAAGCCACTTCCTTACCCA      360
                    ************** *********************************************

NM_001100349.1      AATCTGGACACCTTCTTAGATGATATGAATTTTTTACTTGCTTTAATTGCCCAAGGACCT      420
H                   AATCTGGACACCTTCTTAGATGATATGAATTTTTTACTTGCTTTAATTGCCCAAGGACCT      420
E                   AATCTGGACACCTTCTTAGATGATATGAATTTTTTACTTGCTTTAATTGCCCAAGGACCT      420
                    ************************************************************

NM_001100349.1      GTTAAGACCTATACCCATCGGCGCTTGAAGTTTCTTTCCTCCAAGTTCCAGGTCCATCAG      480
H                   GTTAAGACCTATACCCATCGGCGCTTGAAGTTTCTTTCCTCCAAGTTCCAGGTCCATCAG      480
E                   GTTAAGACCTATACCCATCGGCGCTTGAAGTTTCTTTCCTCCAAGTTCCAGGTCCATCAG      480
                    ************************************************************

NM_001100349.1      ATGCTCAACGAGATGGATGAGT      502
H                   ATGCTCAACGAGATGGATGAGT      502
E                   ATGCTCAACGAGATGGATGAGT      502
                    **********************
```

Figure S12. Assessment of healthy and endometritis dairy cows with sequences derived from GenBank gb|NM_001100349.1| and *AMPD1* marker (502-bp), indicating nitrogenous bases matching for DNA.
